# Editorial: Novel technologies and methods for monitoring exogenous harmful residues in traditional and local medicinal plants and fungi

**DOI:** 10.3389/fphar.2026.1819582

**Published:** 2026-04-30

**Authors:** Bing Wang, Yuanyuan Ge, Ping Wang, Shuhong Wang, Xie-an Yu

**Affiliations:** NMPA Center for Innovation and Research in Regulatory Science, Shenzhen Institute for Drug Control, Shenzhen, China

**Keywords:** fungi, local medicinal plants, monitoring exogenous harmful residues, new regulation, TCM

Exogenous hazardous substances in the safety control of traditional and local medicinal plants and fungi mainly include heavy metals, pesticide residues, sulfur dioxide and mycotoxins. These substances accumulate in them, affecting growth, changing components and endangering ecological stability. Improper harvesting, processing, storage and transportation also cause contamination, threatening patient health and hindering international recognition of TCM. Thus, limit testing of these toxicants is necessary to ensure clinical safety (Chen et al., 2023).

Currently, exogenous toxic substances in medicinal plants and fungi are limited-tested per pharmacopoeial standards after pretreatment. Heavy metals, pesticides, mycotoxins, and sulfur dioxide are analyzed by ICP-MS, GC-MS/MS, LC-MS/MS, HPLC-MS/MS, titration, or ion chromatography, with results compared to legal limits (National Pharmacopoeia Commission, 2025). However, these conventional methods are generally limited by several drawbacks, including high instrumental costs, complicated sample pretreatment procedures, long analysis times, and stringent requirements for operator expertise, thereby restricting their widespread applicability in the fields, at the planting bases, in the medicinal product markets, during production, circulation, and even in clinical settings.

In response to this issue, 50 authors have submitted six articles on this topic to provide technical and methodological support for scientific regulation. Xu et al. summarized sulfur fumigation effects, detection methods and proposed key suggestions for TCM processing and standards. Ge et al. developed a novel method for the determination of sulfur dioxide residues in the wolfberry using a fluorescent probe, and verified its reliability by comparison with the pharmacopoeial method. Lai et al. developed a novel sensor method based on AB-Cu NPs for the screening of organophosphorus pesticide residues in TCM. Liu et al. proposed an “electrostatic adsorption-immobilised probe and dual-enzyme cascade” strategy to construct a novel condensation-driven fluorescent sensor for organophosphorus pesticide residue detection. Guo et al. described in detail the detection bottlenecks and solutions for exogenous contaminants in TCM, and proposed a unified system integrating contaminant detection and degradation. Li et al. developed a rapid H1R antagonist identification method using BODIPY FL histamine, laying a foundation for subsequent exogenous toxicity detection.

Studies have shown that the combination of fluorescent sensing technology based on functionalized carbon dots (CDs) and machine learning can identify and detect various metal ions (Zhang et al., 2026). An advanced electrochemical sensor has been developed for the simultaneous detection of Cd^2+^, Pb^2+^, Cu^2+^, and Hg^2+^ ions (Madhivanan et al., 2025). Some studies have reported a surface-enhanced Raman spectroscopy platform combining Au@Ag core-shell nanocrystals (Au@AgCNCs) with machine learning for rapid screening and identification of multi-target pesticide residues in fruit juices (Jiang et al., 2026). Although the detection of multiple mycotoxins in maize using fluorescence spectroscopy combined with machine learning has been reported in literature, its limitation lies in the lack of confirmation with authentic positive samples (Venturini et al., 2026). A fluorescent probe based on fluorescence resonance energy transfer has been reported in literature, with a rapid and sensitive method established for the detection of sulfur dioxide and its derivatives (Li et al., 2026).

In summary, various novel analytical methods have been applied for the detection of exogenous toxic substances, including fluorescent probes, optoelectronic sensing, and electrochemical techniques, Surface-enhanced Raman spectroscopy and machine learning methods. These new technologies and methods exhibit advantages including simple operation, no requirement for sophisticated instruments, high detection sensitivity, rapid detection speed, low demand for personnel expertise, and good portability, showing great potential for on-site rapid detection. However, due to the complexity of sample matrices, these new technologies and methods face several challenges in practical regulatory on-site detection. These include strong matrix interference in some special TCMs, relatively high false-positive and false-negative rates, and relatively low sensitivity. Their stability, service life, and accuracy require repeated validation; visual detection of some samples is susceptible to interference; and these detection methods cannot be universally applied to different types of samples.

**FIGURE 1:**
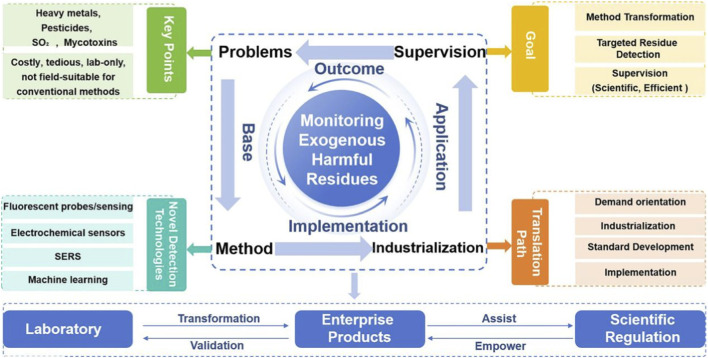
Schematic diagram of Editorial.

Thus, technology translation from the lab to regulatory sites faces “last-mile” challenges in stability, anti-interference and standardization. Focusing on demand orientation, industrialization, standard development and collaborative application, this issue explores pathways for novel detection technologies to become official regulatory tools, aiming to build a full-chain quality safety system and support the high-quality development and globalization of TCM (Figure 1).

Identify regulatory pain points to align technologies with actual needs and avoid disconnection. It is necessary to systematically sort out regulatory priorities for exogenous toxic substances, so as to select detection technologies characterized by stability, portability, sensitivity, anti-interference capability and matrix adaptability. A research-regulator-enterprise collaboration with clearly defined roles should be established.

In light of the selected high-performance technologies and the demand for portable, rapid on-site supervision, instrumental transformation can be implemented. For example, fluorescent probes and fluorescence sensing can be integrated with paper-based substrates, microfluidic chips, and smartphones to develop portable fluorescent test strips and handheld fluorescence detectors. Electrochemical techniques can be miniaturized into portable electrochemical detectors, simplifying operation, reducing reliance on professional personnel, and enabling rapid on-site detection without sophisticated instruments.

Based on the newly developed regulatory tools, corresponding novel detection methods are formulated, with clear testing procedures, operational protocols and result judgment criteria, to address practical issues including false positives/negatives, insufficient sensitivity and matrix interference. As standards are the core basis for regulatory enforcement, validated methods should be upgraded to regulatory standards, and a hierarchical, classified and comprehensive standard system should be established.

A demonstration and promotion mechanism will be established, with pilot applications in key areas, and supporting policies improved to boost industrialization and lower costs. A technical support platform and feedback mechanism will be built to provide follow-up services and enable iterative optimization for TCM detection.

This thematic study facilitates the translation of laboratory analytical methods into field-deployable standards, enabling science-driven, high-efficiency supervision with strong practicality and scalability.
